# Quantification of Sepsis Model Alerts in 24 US Hospitals Before and During the COVID-19 Pandemic

**DOI:** 10.1001/jamanetworkopen.2021.35286

**Published:** 2021-11-19

**Authors:** Andrew Wong, Jie Cao, Patrick G. Lyons, Sayon Dutta, Vincent J. Major, Erkin Ötleş, Karandeep Singh

**Affiliations:** 1 Department of Internal Medicine, University of Michigan Medical School, Ann Arbor; 2 Department of Computational Medicine and Bioinformatics, University of Michigan Medical School, Ann Arbor; 3 John T. Milliken Department of Medicine, Washington University School of Medicine, St Louis, Missouri; 4 Department of Emergency Medicine, Massachusetts General Hospital, Boston; 5 Department of Population Health, New York University Grossman School of Medicine, New York; 6 Medical Scientist Training Program, University of Michigan, Ann Arbor; 7 Department of Industrial and Operations Engineering, University of Michigan, Ann Arbor; 8 Department of Learning Health Sciences, University of Michigan Medical School, Ann Arbor; 9 School of Information, University of Michigan, Ann Arbor

## Abstract

This descriptive study evaluates the association between nursing reports of sepsis overalerting and alert volume by quantifying the number of alerts generated by the Epic Sepsis Model at 24 US hospitals before and during the COVID-19 pandemic.

## Introduction

Sepsis early warning systems aim to assist clinicians in recognizing and treating sepsis. Historically, these early warning systems have relied on simple clinical rules, such as systemic inflammatory response syndrome criteria, to identify patients with possible sepsis. To date, sepsis early warning systems have not been shown to reliably improve patient outcomes,^1^ and artificial intelligence (AI) systems such as the widely implemented Epic Sepsis Model (ESM) are beginning to replace them.

Concerns have arisen recently regarding the potential for sepsis models to cause alert fatigue.^2,3,4^ Between 3 and 4 weeks after its first COVID-19 hospitalization, the University of Michigan paused ESM-generated alerts in April 2020 after nursing reports of overalerting.^5^

This increase in alerting could have resulted from dataset shift, a phenomenon in which model performance deteriorates as a result of changes in the case mix (eg, COVID-19).^5^ However, even accurate alerts can be disruptive in the presence of resource constraints. For example, more than a quarter of hospitalized patients in the first wave of the COVID-19 pandemic in 2020 required a transfer to an intensive care unit or died.^6^

We quantified the number of alerts generated by the ESM at 24 hospitals in the months before and during the COVID-19 pandemic to (1) evaluate the extent to which nursing reports of sepsis overalerting were linked to the alert volume and (2) examine the variation in alert volume across US hospitals.

## Methods

This descriptive study was approved by the institutional review boards at the University of Michigan, Washington University, and Mass General Brigham and was considered to be nonregulated at the New York University Grossman School of Medicine. The need for consent was waived because the research involved no more than minimal risk to participants, the research could not be carried out practicably without the waiver, and the waiver would not adversely affect the rights and welfare of the participants.

ESM scores were calculated prospectively from 24 hospitals across 4 geographically diverse health systems (University of Michigan in Ann Arbor, Michigan; New York University Langone Health in New York, New York; Mass General Brigham in Boston, Massachusetts; and BJC HealthCare in St Louis, Missouri) from November 3, 2019, to April 25, 2020. These scores were aligned to the hospitals’ first case of COVID-19. We compared the total hospital census, the proportion of patients generating sepsis alerts, and the frequency of sepsis alerts using data before and during the COVID-19 pandemic, with an alerting threshold of 6 and a maximum of 1 alert per patient per day. Further details are provided in the eMethods in the Supplement.

## Results

In the 3 weeks before and after the first case of COVID-19 in each US health system in this study, the proportion of patients generating sepsis alerts per day more than doubled from 9% (953 of 10 159) to 21% (1363 of 6634), respectively (Table). However, the total hospital census declined by 35% (from 10 159 to 6634). The total number of alerts per day increased by 43% (from 953 to 1363) despite the lower hospital census.

**Table.** Comparison of Total Hospital Census, Total Number of Alerts per Day, and Proportion of Patients Generating Alerts Before and After Hospitals’ First Case of COVID-19^a^

No. of days after the first case of COVID-19	No. of patients in the total hospital census	No. of alerts per day	Proportion of patients generating alerts, %^b^
–56 (to –48)	10 302	881	9
–49	10 105	863	9
–42	10 183	899	9
–35	10 364	936	9
–28	10 245	983	10
–21	10 159	953	9
–14	10 001	956	10
–7	10 189	894	9
0	9830	921	9
7	8593	911	11
14	6861	1042	15
21	6634	1363	21
28	6475	1478	23
35 (to 41)	6536	1504	23

^a^ Data were obtained from each calendar week and are reported at the daily level; day 0 represents the calendar week of the first case of COVID-19, beginning with day 0 and ending at day 6.

^b^ The proportion of patients generating alerts represents the number of alerts per day divided by the total hospital census.

Larger hospitals generally experienced an increase in the proportion of patients generating sepsis alerts, whereas the change in the alerting proportion was more heterogeneous across smaller hospitals (Figure). The pausing of sepsis alerts at the University of Michigan corresponded with the increase in alerting (Figure).

**Figure.**
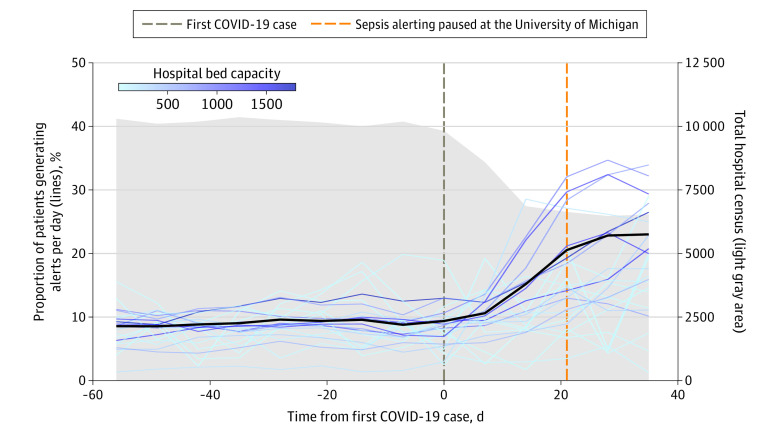
Comparison of Total Hospital Census and Proportion of Patients Generating Sepsis Alerts at Each Hospital, Aligned by Hospitals’ First Case of COVID-19 Each hospital is depicted by a line; darker lines indicate higher hospital bed capacity. The thick black line represents the average across all hospitals. Calculations are averaged for each calendar week; day 0 represents the calendar week of the first case of COVID-19, beginning with day 0 and ending at day 6. The vertical dashed brown line depicts day 0, and the vertical dashed orange line depicts the day the Epic Sepsis Model was paused at the University of Michigan. Further details are provided in the eMethods in the Supplement.

## Discussion

Although the increase in the proportion of patients generating sepsis alerts in this study can be explained by the cancellation of elective surgeries and a higher average acuity among the remaining hospitalized patients, the 43% increase in total alerts illustrates the increased alerting burden imposed by COVID-19 on a sepsis model.

Our study was limited in that we did not evaluate the model’s accuracy. However, even if the alerts were accurate, many existing sepsis workflows are built around bacterial sepsis and thus may not be entirely appropriate in the context of COVID-19.

Being able to rapidly assess and disable AI-based alerts is a responsibility faced by health systems using AI to support clinical care. Given the susceptibility of AI-based systems to changes in alerting patterns, clinical AI governance within health systems may play a role in monitoring and supporting deployed AI systems.

## Notes

Supplement.
eMethods.

eReferences
