# Supplementary material for: Quantification of Sepsis Model Alerts in 24 US Hospitals Before and During the COVID-19 Pandemic
Source: JAMA Netw Open. 2021 Nov 19;4(11):e2135286. doi: 10.1001/jamanetworkopen.2021.35286 (PMC8605481; doi:10.1001/jamanetworkopen.2021.35286)
Supplement: Supplement. — eMethods. eReferences [file jamanetwopen-e2135286-s001.pdf]

# Supplementary Online Content

Wong A, Cao J, Lyons PG, et al. Quantification of sepsis model alerts in 24 US hospitals before and during the COVID-19 pandemic. *JAMA Netw Open*. 2021;4(11):e2135286. doi:10.1001/jamanetworkopen.2021.35286

**eMethods.**
**eReferences**

This supplementary material has been provided by the authors to give readers additional information about their work.

© 2021 Wong A et al. *JAMA Network Open*.

**eMethods.**

Rationale for Epic Sepsis Model (ESM) Threshold of 6

In a recent single-center evaluation, the ESM was found to generate alerts in 18% of hospitalized patients at the University of Michigan (during a “silent” evaluation using an alerting threshold of 6) when examining data from 2018 to 2019 while missing 67% of sepsis cases (i.e., a sensitivity of 33%), where sepsis was defined by a composite of the Centers for Disease Control and Prevention surveillance criteria and (2) the Centers for Medicare and Medicaid Services SEP-1 Criteria.

This same threshold of 6 was selected in this study for three reasons. First, it is within the range (5-8) recommended by the model developer.[1] Second, multiple preprints, conference proceedings, and publications evaluating the ESM have relied on thresholds of 5 or 6.[1–4] And third, it is the threshold that has been in clinical use to generate alerts at the University of Michigan.[1] As compared to an ESM threshold of 5, a threshold of 6 would be expected to generate fewer alerts and thus is a more conservative choice.

Calculation of the Number of Total Alerts and Proportion of Patients Generating Alerts

At each hospital included in this analysis, the ESM runs at intervals between 15-20 minutes on patients in the emergency department and inpatient settings, including both intensive care unit (ICU) and non-ICU settings. Using an alerting threshold of 6, we calculated the number of patients in whom the ESM would have generated alerts based on prospectively calculated ESM scores. Alerts were generated on three inpatient units at the University of Michigan during this time period, though ESM scores were calculated for all adult emergency department and inpatient settings. Although no actual alerts were generated at New York University Langone Health, Mass General Brigham, and BJC HealthCare during this time, the prospective calculation of ESM scores at these institutions allowed us to calculate the number of alerts that would have been generated.

Assuming that alerts would be muted and thus could only be generated at most once per day for a given patient even if they exceeded the alerting threshold multiple times, we first calculated how many alerts would have been calculated per each calendar week (Sunday to Saturday) for patients, with a maximum of 7 alerts per week possible for each patient. We then calculated the mean number of alerts per day by dividing the total number of weekly alerts by 7. To calculate the proportion of patients generating an alert, we divided the number of patients generating an alert by the number of patients scored by the ESM.

Calculation of Hospital Census

The **Figure** depicts the total number of patients evaluated by the ESM across the 24 hospitals in our study. While this is labeled “hospital census,” this number is actually larger than the actual hospital census because it includes all patients for whom the ESM was calculated (and thus were alert-eligible), including patients evaluated in the emergency department who were discharged.

© 2021 Wong A et al. *JAMA Network Open*.

Calculation of Individual Hospital Bed Capacity

The **Figure** color-codes the hospitals by size. While this is labeled as “individual hospital bed capacity,” this number is actually larger than the actual hospital bed capacity because it includes the maximum number of patients in whom the ESM was calculated on any given day during the study period, including patients evaluated in the emergency department who were discharged.

Timing of COVID-19 Onset

The hospitals’ first case of COVID-19 (day zero in the **Figure**) was determined at the level of the health system, and not individually calculated at each hospital. If the first case occurred during the calendar week, we assumed that the date of onset was actually the beginning of that calendar week (because the data depicted in the Figure is averaged at the weekly level). For Mass General Brigham, the date of COVID-19 onset we used was March 1, 2020. For the University of Michigan and New York University Langone Health, the date of COVID-19 onset we used was March 8, 2020. For BJC HealthCare, the date of COVID-19 onset we used was March 15, 2020.

Hospitals Included in the Analysis

Below is a complete list of the hospitals included in our analysis.

*University of Michigan*

University of Michigan Hospital, Ann Arbor, MI

*Mass General Brigham*

Brigham and Women's Faulkner Hospital, Jamaica Plain, MA
Brigham and Women's Hospital, Boston, MA
Cooley Dickinson Hospital, Northampton, MA
Massachusetts General Hospital, Boston, MA
Martha's Vineyard Hospital, Oak Bluffs, MA
Nantucket Cottage Hospital, Nantucket, MA
North Shore Medical Center, Salem, MA
Newton-Wellesley Hospital, Newton, MA
Wentworth-Douglass Hospital, Dover, NH

*New York University Langone Health*

NYU Langone Hospital--Brooklyn, Brooklyn, NY
NYU Langone Hospital--Long Island, Long Island, NY
Tisch Hospital and Kimmel Pavilion, New York, NY
NYU Langone Orthopedic Hospital, New York, NY

*BJC HealthCare*

Alton Memorial Hospital, Alton, IL
Barnes-Jewish Hospital, St. Louis, MO
Barnes-Jewish St. Peters Hospital, St. Peters, MO
Barnes-Jewish West County Hospital, Creve Coeur, MO

© 2021 Wong A et al. *JAMA Network Open*.

Missouri Baptist Medical Center, St. Louis, MO
Missouri Baptist Sullivan Hospital, Sullivan, MO
Parkland Health Centers, Farmington, MO
Progress West Healthcare Center, O'Fallon, MO
Boone Hospital, Columbia, MO
Christian Hospital, St. Louis, MO

**eReferences**


1. Wong A, Otles E, Donnelly JP, et al. External Validation of a Widely Implemented Proprietary Sepsis Prediction Model in Hospitalized Patients. *JAMA Intern Med*. 2021;181(8):1065-1070.

2. Cull J, Blackhurst D, Kothari S, et al. 1235: Validating the Epic Sepsis Inpatient Predictive Analytic Tool as a Sepsis Alert System. *Crit Care Med*. 2021;49(1):621.

3. Bennett T, Russell S, King J, et al. Accuracy of the epic sepsis prediction model in a regional health system. *arXiv [statAP]*. Published online February 19, 2019. http://arxiv.org/abs/1902.07276

4. Lyons PG, Ramsey B, Simkins M, Maddox TM. How Useful Is the Epic Sepsis Prediction Model for Predicting Sepsis? In: *TP14. TP014 DIAGNOSTIC AND SCREENING INSIGHTS IN PULMONARY, CRITICAL CARE, AND SLEEP*. American Thoracic Society International Conference Abstracts. American Thoracic Society; 2021:A1580-A1580.


© 2021 Wong A et al. *JAMA Network Open*.
